# Enhanced expression of membrane proteins in *E. coli *with a *P*_BAD _promoter mutant: synergies with chaperone pathway engineering strategies

**DOI:** 10.1186/1475-2859-10-105

**Published:** 2011-12-09

**Authors:** Brent L Nannenga, François Baneyx

**Affiliations:** 1 Department of Chemical Engineering, University of Washington, Seattle, WA 98195-1750, USA

## Abstract

**Background:**

Membrane proteins (MPs) populate 20-30% of genomes sequenced to date and hold potential as therapeutic targets as well as for practical applications in bionanotechnology. However, MP toxicity and low yields in normally robust expression hosts such as *E. coli *has curtailed progress in our understanding of their structure and function.

**Results:**

Using the seven transmembrane segments *H. turkmenica *deltarhodopsin (HtdR) as a reporter, we isolated a spontaneous mutant in the arabinose-inducible *P*_BAD _promoter leading to improved cell growth and a twofold increase in the recovery of active HtdR at 37°C. A single transversion in a conserved region of the cyclic AMP receptor protein binding site caused the phenotype by reducing *htdR *transcript levels by 65%. When the mutant promoter was used in conjunction with a host lacking the molecular chaperone Trigger Factor (Δ*tig *cells), toxicity was further suppressed and the amount of correctly folded HtdR was 4-fold that present in the membranes of control cells. More importantly, while improved growth barely compensated for the reduction in transcription rates when another polytopic membrane protein (*N. pharonis *sensory rhodopsin II) was expressed under control of the mutant promoter in wild type cells, a 4-fold increase in productivity could be achieved in a Δ*tig *host.

**Conclusions:**

Our system, which combines a downregulated version of the tightly repressed *P*_BAD _promoter with a TF-deficient host may prove a valuable alternative to T7-based expression for the production of membrane proteins that have so far remained elusive targets.

## Background

Membrane proteins (MPs) play pivotal roles in a variety of cellular functions, many of which are essential to survival [1]. Despite their physiological importance, the study of MPs is lagging due largely to the fact that they are difficult to express in a functional form and at levels needed for biochemical and structural studies. *Escherichia coli *is a popular host for MP overexpression due to its well understood genetics and rapid growth [2]. However, as with other expression systems, high-level MP production is typically toxic to the cell and the yields of biologically active material are generally poor.

Based on the observation that the overexpression of MPs in *E. coli *leads to their aggregation and to reduced levels of host membrane and secretory proteins [3], it has been suggested that MP toxicity is due to the overloading of the Sec-dependent translocation machinery which handles both the post-translational export of secretory proteins and the co-translational insertion of most inner MP into the lipid bilayer [4]. Recently, we have shown that eliminating the signal recognition particle (SRP) -Trigger Factor (TF) competition by making use of TF-deficient (Δ*tig*) expression strains can significantly improve the accumulation of functional MP in the bacterial inner membrane [5]. A more common approach, however, has been to make use of *E. coli *C41(DE3) and C43(DE3) [6], two BL21(DE3) derivatives containing a mutation in the *lac*UV5 promoter that decreases the production of chromosomally-encoded T7 RNA polymerase, and hence the transcription rate of MP genes cloned downstream of the T7 promoter [7]. The same net effect can be achieved by making use of plasmids co-expressing T7Lys (e.g. pLemo, pLysS, pLysE) [7,8], a T7 RNA polymerase inhibitor that reduces the overall transcription rates of genes placed under T7 promoter control.

In addition to being too strong for MP expression, a drawback of the T7 promoter is its lack of tight repression in the absence of inducer, which may be problematic since even basal levels of MPs can be toxic to the cell [6,8]. The arabinose-inducible *P*_BAD _promoter of the *araBAD *operon [9] is a moderately strong and tightly repressed promoter that has been successfully used for producing MPs in *E. coli *[5,10-12]. *P*_BAD _is negatively regulated by AraC when no L-arabinose is present in the medium, and it is positively regulated by both AraC in the presence of arabinose and the cyclic AMP (cAMP) receptor protein (CRP, also known as CAP, catabolite gene activator protein) in the absence of glucose [13]. When loaded with cAMP, the CRP homodimer binds to a consensus sequence located upstream of more than 100 *E. coli *promoters [14]. This binding favors transcription initiation [15] both by bending the DNA [16,17] and by recruiting RNA polymerase to facilitate its interaction with the core promoter [18].

Here, we describe the isolation of a single nucleotide transversion in one of the CRP interaction domains of the *P*_BAD _promoter that reduces the transcription rates by about 70% and can improve the yields of polytopic MPs, particularly when combined with a chaperone pathway reprogramming strategy relying on the use of Trigger Factor (TF) deficient mutants [5].

## Results and Discussion

### Isolation of a mutant *P*_BAD _expression plasmid that alleviates HtdR toxicity

The archaeal rhodopsin *Haloterrigena turkmenica *deltarhodopsin (HtdR) is a light-driven outwards proton pump that binds the chromophore all-*trans *retinal and belongs to the G-protein coupled receptor (GPCR) super family of 7 transmembrane (TMS) segment MPs. Retinal-bound and properly folded HtdR confers the cell membrane a characteristic purple color due to an adsorption spectrum that exhibits a strong maximum at ≈ 550 nm [19]. We sought to take advantage of this phenotype to identify *E. coli *mutants that were more efficient at functional MP expression. In initial screen design experiments, TF-deficient (Δ*tig*) cells [20] harboring plasmid pHtdR200, a ColE1 derivative encoding a hexahistidine-tagged version of the *htdR *gene under transcriptional control of the *P*_BAD _promoter [5], were plated onto LB agar plates supplemented with L-arabinose and all-*trans *retinal and incubated for 36 h at 37°C. While most colonies were small and a light shade of red due to the toxicity of HtdR overexpression, we isolated a spontaneous mutant that was both large and purple.

**Figure 1:**
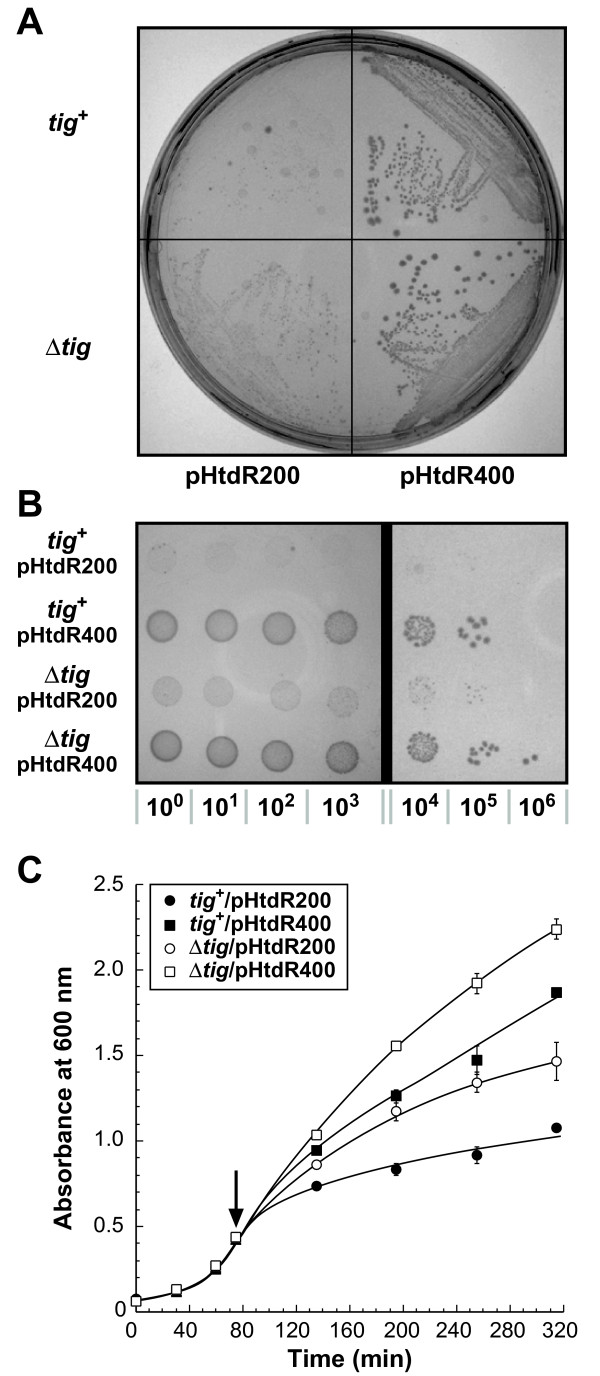
**A mutant plasmid improves the growth and viability of cells expressing HtdR**. The growth of cells harboring pHtdR200 or pHtdR400 was examined by streaking (A) or spot assays (B) on LB agar plates supplemented with 50 μg/mL kanamycin, 100 μM of all-*trans *retinal, and 0.2% L-arabinose. (C) Wild type (*tig*^+^, closed symbols) and TF-deficient cells (Δ*tig*, open symbols) harboring the indicated plasmids were grown in LB-kanamycin at 37°C and HtdR synthesis was induced by addition of 0.2% L-arabinose in mid-exponential phase (arrow).

To determine if the apparent increase in functional HtdR production was associated with a mutation in the expression vector or in the chromosomal DNA, the plasmid was isolated, named pHtdR400, and re-transformed into fresh, isogenic wild type and Δ*tig *cells. Figure 1A shows that pHtdR400 alone was sufficient to confer healthy growth to both wild type and Δ*tig *cells that had been incubated overnight on LB-arabinose plates. In fact, under conditions of HtdR overexpression, the viability of *tig*^+ ^pHtdR400 cells was improved by 5 orders of magnitude relative to that of pHtdR200 transformants (Figure 1B) and their specific growth rate in liquid culture by 60% (Figure 1C, closed symbols). Use of the pHtdR400 plasmid also enhanced the fitness of Δ*tig *mutants: viability increased 100-fold and specific growth rates by 30% relative to control cells (Figure 1B-C).

### Impact on functional HtdR expression and productivity

**Figure 2:**
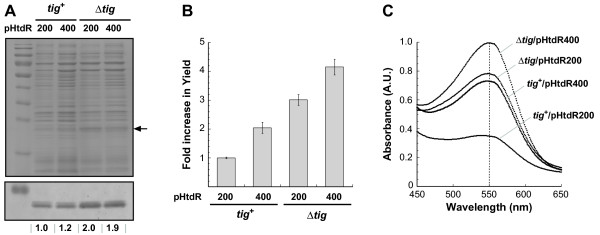
**Combining pHtdR400-based expression with the use of TF-deficient strains leads to large gain in HtdR productivity**. (A) Membrane fractions corresponding to identical numbers of cells were collected 3 h post-induction and analyzed for HtdR content by SDS-PAGE (top) or immunoblotting (bottom) with anti-His antibodies. The arrow shows the migration position of HtdR while numbers below the blot quantify the intensity of each band relative to an arbitrary value of 1 for control cells. (B) Influence of plasmid and genetic background on the total yield of HtdR 3 h post-induction. (C) Absorption spectra of solubilized membranes isolated 3 h post-induction from the indicated cells.

We next asked if the improved growth characteristics of pHtdR400 transformants would translate into higher levels of target protein expression. To address this question, the various cultures were grown to mid-exponential phase in LB medium and at 37°C, HtdR synthesis was initiated by addition of 0.2% L-arabinose, and cells were harvested 3 h post-induction. As expected from our previous work [5], the use of Δ*tig *cells led to a twofold increase in the amount of membrane-integrated HtdR expressed from pHtdR200. By contrast, the overall levels of deltarhodopsin were comparable in pHtdR200 and pHtdR400 transformants, whether or not the host strain was wild type or *tig *deficient (Figure 2A). However, when the improved growth of pHtdR400 transformants was taken into account, the yield of HtdR doubled in wild type cells. More importantly, the beneficial effects of pHtdR400-driven expression and chromosomal TF inactivation could be combined, leading to a more than fourfold increase in HtdR productivity relative to *tig*^+ ^pHtdR200 control cultures (Figure 2B) and to shake flask yields as high as 20 mg/L of culture.

We took advantage of the fact that the optical signature of retinal-bound HtdR - and more specifically of its absorption maximum at 550 nm - is a sensitive reporter of proper folding [5,19] to estimate how much of the protein was functional in the membranes of cells harvested 3 h post-induction. In very good agreement with the results of Figure 2B, the intensity of the 550 nm peak in *tig*^+ ^pHtdR400 membrane fractions was twice that measured in *tig*^+ ^pHtdR200 samples, and there was an about 30% increase in 550 nm absorption when membranes from Δ*tig *pHtdR400 cells were compared to those from Δ*tig *pHtdR200 cells (Figure 2C). We conclude that the additional deltarhodopsin produced in pHtdR400 transformants is properly folded. (Note that intensities at 550 nm cannot be directly compared between *tig*^+ ^and Δ*tig *cells since TF inactivation changes the levels of expression of host MP and thus the optical characteristics of the membrane.)

To summarize, under the selective pressure of HtdR expression, pHtdR400 has acquired one or several mutations that significantly improve cell growth and functional HtdR productivity, but do not increase the accumulation of the MP on a per-cell basis as TF-inactivation does.

### Identification of the mutation and analysis of its effects

**Figure 3:**
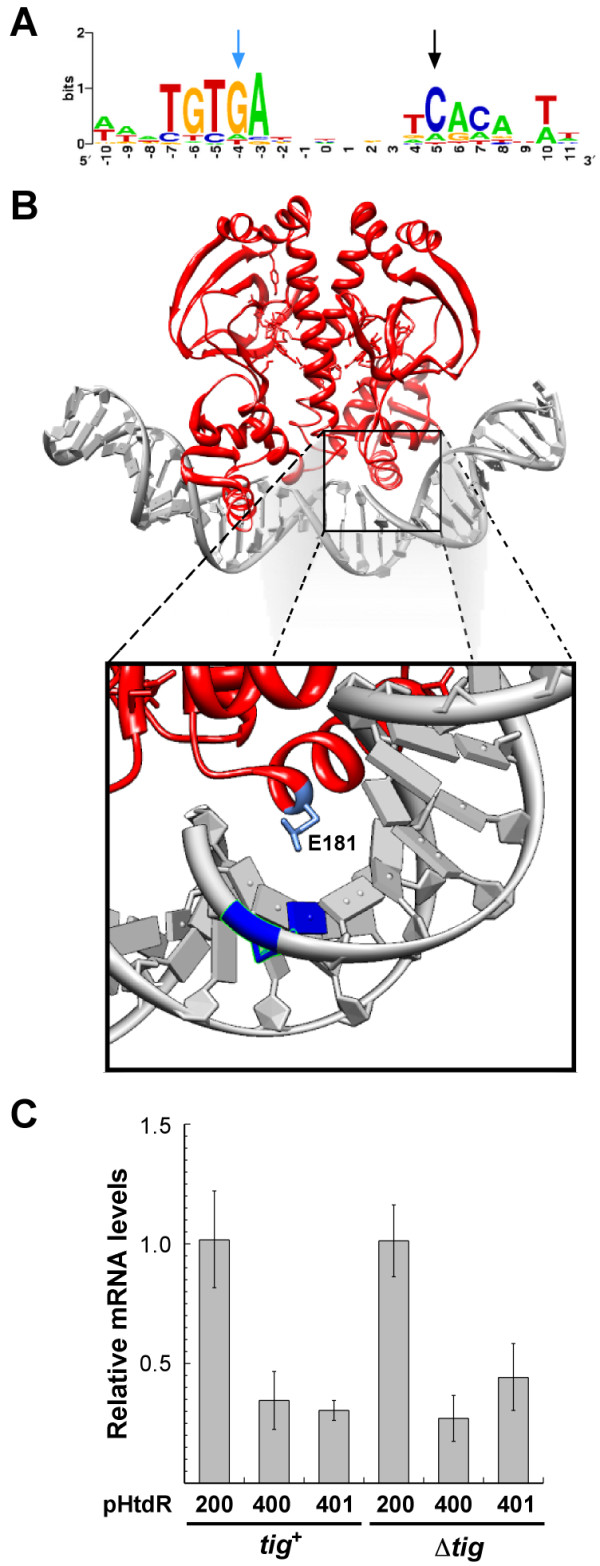
**Plasmid pHtdR400 carries a single nucleotide mutation in the CRP binding site of the P_BAD _promoter that strongly reduces *htdR *transcription**. (A) Sequence logo [29] from the alignment of 59 CRP binding sites [21,30]. The size of each nucleotide is proportional to its degree of conservation. The black arrow shows the location of the cytosine to adenosine mutation in pHtdR400. The blue arrow shows the location of the guanine to thymine mutation introduced in pHtdR401. (B) Ribbon structure of CRP bound to its binding site rendered with Chimera using 1CGP coordinates [17]. The box shows the proximity of Glu^181 ^to the cytosine to adenosine mutation (blue) in pHtdR400. (C) Relative mRNA levels were quantified from the indicated cells 1 h post-induction.

Plasmid pHtdR400 was sequenced to shed light on the mechanism(s) responsible for the improvement in HtdR productivity. We identified a single cytosine to adenosine mutation located 98-nt from the transcription start site of the *P*_BAD _promoter and mapping in a highly conserved region of the 22 bp-long and symmetrical consensus CRP binding site (Figure 3A, black arrow) [21]. To confirm that it was responsible for the improvement in HtdR productivity, the transversion was introduced into pHtdR200 via site directed mutagenesis. The growth and HtdR expression patterns of wild type and Δ*tig *cells transformed with this plasmid (pHtdR400bis) were indistinguishable from those of pHtdR400 transformants.

Because the mutation occurs near the primary kink of the bended CRP-DNA complex [17] and affects a contact with Glu181 [22] which is critical for CRP binding [23,24] (Figure 3B), we suspected that it would affect positive regulation and lead to a decrease in transcription rates. To test this hypothesis, we used real-time quantitative polymerase chain reaction (RT-qPCR) to measure the relative levels of *htdR *mRNA in *tig*^+ ^and Δ*tig *cells harboring either pHtdR200 or pHtdR400. Figure 3C shows that the mutation led to ≈ 65% reduction in *htdR *transcript concentration irrespective of the genetic background.

To further confirm that inefficient CRP binding was responsible for the decrease in transcription rates, we took advantage of the fact that the CRP homodimer binds to a consensus sequence with twofold symmetry and introduced a guanine to thymine mutation in the opposite half-site of pHtdR200, in essence building the counterpart of the spontaneous cytosine to adenine mutation (Figure 3A, blue arrow). The resulting plasmid (pHtdR401) was introduced into wild type and Δ*tig *cells and RT-qPCR experiments repeated. Figure 3C shows that *htdR *mRNA levels produced from pHtdR401 were virtually identical to those produced from pHtdR400 in both genetic backgrounds.

In an argument similar to that invoked for explaining the improved performance of C41(DE3) and C43(DE3) strains [7], we conclude that the net reduction in mRNA levels caused by inefficient CRP-dependent activation of the *P*_BAD _promoter decreases the flux of HtdR to the inner membrane and alleviates Sec translocon overloading by providing a better match between the supply of incoming MPs and the insertional capacity at the membrane. This in turns reduces toxicity, improves cell growth, and leads to higher productivities.

It is also worth noting that a 2/3 reduction in *htdR *transcription does not correlate with a net decrease in the amount of membrane-integrated HtdR on a per-cell basis (Figure 2A). We believe that this result is protein-specific (see below) and that there is a "sweet spot" for which the transcription rate of a target MP is well matched with the rate at which the translocational machinery can insert and/or fold this particular substrate in the lipid bilayer.

### Reduced transcription and TF inactivation synergistically improve Sensory Rhodopsin II production

To investigate whether the yield improvements observed with HtdR could be generalized to other MPs, we constructed pBLN400 a derivative of our standard pBLN200 *P*_BAD _expression vector incorporating the cytosine to adenosine mutation in its CRP binding site. We next placed a different His-tagged rhodopsin, *Natronobacterium pharonis *sensory rhodopsin II (pSRII) [25], under control of the mutated promoter and transformed the resulting plasmid (pPPR400) into both wild type and *tig *null cells.

**Figure 4:**
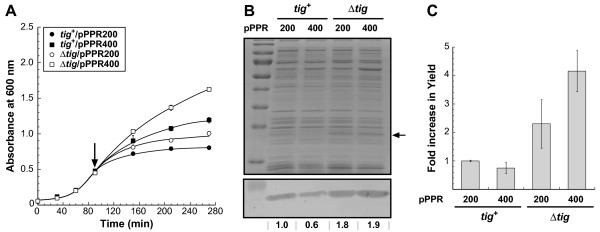
**Reduced transcription and TF-inactivation synergistically improve the yields of pSRII**. (A) Wild type (*tig*^+^, closed symbols) and TF-deficient cells (Δ*tig*, open symbols) harboring the indicated plasmids were grown in LB-kanamycin at 37°C and pSRII synthesis was induced by addition of 0.2% L-arabinose in mid-exponential phase (arrow). (B) Membrane fractions corresponding to identical numbers of cells were collected 3 h post-induction and analyzed for pSRII content by SDS-PAGE (top) or immunoblotting (bottom) with anti-His antibodies. The arrow shows the migration position of pSRII while numbers below the blot quantify the intensity of each band relative to an arbitrary value of 1 for control cells. (C) Influence of plasmid and genetic background on the total yield of pSRII 3 h post-induction.

Consistent with what would be expected from a weaker promoter, *tig*^+ ^pPPR400 cells accumulated about 40% less pSRII in their membranes compared to pPPR200 transformants (Figure 4B), but healthier cell growth (Figure 4A, ■) led to nearly identical total yields (Figure 4C). Also as expected [5], Δ*tig *cultures producing pSRII from the standard *P*_BAD _promoter exhibited improved growth (but not as much as *tig*^+ ^pPPR400 cells; compare ■ and ○ in Figure 4A), higher levels of membrane-integrated pSRII (Figure 4B) and over 2-fold higher productivities relative to control cultures (Figure 4C). Expression of pSRII from the mutant promoter in Δ*tig *cells did not lead to a reduction in the amount of membrane-integrated protein as it did in the wild type. Rather, this combination caused a large improvement in cell growth that translated into shake flask yields of 10 mg/L of culture and a more than 4-fold increase in pSRII productivity relative to *tig*^+ ^pPPR200 control cultures (Figure 4). These results closely parallel those obtained with HtdR and indicate that the productivity ceiling that one reaches by decreasing transcription rates can be shattered by combining it with chaperone pathways reprogramming.

## Conclusions

Downregulating gene transcription by decreasing the intracellular levels of the highly processive T7 RNA polymerase is a well-established approach to improve the yields of MPs whose genes have been placed under control of the bacteriophage T7 promoter. The benefits have been explained by a harmonization of translation and membrane insertion which mitigates the toxicity associated with Sec translocon saturation [4]. Here, we describe a single nucleotide mutation in one of the conserved half-sites of the *P*_BAD _promoter's CRP binding region that improves the recovery yields of active HtdR through a similar mechanism. However, as illustrated with the closely related sensory rhodopsin II, it is possible that improved cell growth only barely compensates for reduced transcription, leading to unchanged MP productivity (Figure 4D).

We recently reported that inactivation of TF is an alternative means to alleviate MP toxicity, likely because it allows signal recognition particle (SRP) unimpeded access to nascent TMS emerging from the ribosome and more efficient delivery of ribosome-nascent membrane protein complexes to FtsY (the SRP receptor) which delivers this cargo to the Sec translocon [5]. Here, we further show that the use of Δ*tig *cells can be combined with reduced transcription strategies to achieve synergistic gains in culture fitness and MP productivity. Thus, how nascent MPs are delivered to the translocon is probably as important as how many chains seek engagement by the Sec machinery in determining recombinant MP toxicity and their eventual yields.

## Materials and methods

### Strains, plasmids and culture conditions

*E. coli *BW25113 [Δ*(araD-araB)567 *Δ*lacZ4787 (::rrnB-3) λ^- ^rph-1 *D*(rhaD-rhaB)568, hsdR514*] [26], KTD101 [BW25113 Δ*tig100*] [20], and plasmid pHtdR200 [5] have been described previously. KTD101(pHtdR200) cells were grown to *A*_600 _≈ 0.45 in 125 mL flasks containing 25 mL of LB media supplemented with 50 μg/mL kanamycin. Samples were streaked on LB agar plates supplemented with 50 μg/mL kanamycin, 100 μM of all-*trans *retinal (Sigma; 10 mM stock solution in methanol), and 0.2% L-arabinose and the plates were incubated at 37°C for 36 h. A colony exhibiting both larger size and pronounced purple color was isolated, grown in LB-kanamycin and the plasmid was purified. The regions of the plasmid corresponding to the *P*_BAD _promoter and regulatory region, origin of replication, *araC *gene and *htdR *gene were sequenced. The mutant plasmid was named pHtdR400 and transformed into chemically competent BW25113 and KTD101. The single nucleotide mutation found in pHtdR400 was introduced into pHtdR200 and pBLN200 [5] by site-directed mutagenesis using primers 5'-CAAAGTGTGACGCCGTGAAAATAATCAATGTGGAC-3' and 5'-GTCCACATTGATTATTTTCACGGCGTCACACTTTG-3', yielding pHtdR400bis and pBLN400, respectively. This mutation was also introduced into the opposite CRP binding half-site of pBLN200 by site-directed mutagenesis using primers 5'-GCTATGGCATAGCAAAGTGTTACGCCGTGCAAATAATCAATG-3' and 5'-CATTGATTATTTGCACGGCGTAACACTTTGCTATGCCATAGC-3' to generate pBLN401. The gene encoding pSRII was excised from pPPR200 [5] using *Nde*I and *Xho*I, and cloned into the same sites of pBLN400 to create pPPR400. The gene encoding HtdR from pHtdR200 was similarly subcloned into pBLN400 to create pHtdR401.

For viability experiments, culture tubes (18 mL) containing 5 mL of LB supplemented with 50 μg/mL kanamycin were inoculated with the indicated cells to an *A*_600 _≈ 0.05 and cells were grown at 37°C to *A*_600 _≈ 0.45. Samples were either streaked directly on LB-agar plates supplemented with 50 μg/mL kanamycin, 0.4% L-arabinose, and 10 μM of all-*trans *retinal, or diluted in LB medium by factors of 10 and spotted on plates in 10 μL aliquots.

### Membrane protein expression, isolation and characterization

HtdR and pSRII were expressed as previously described [5]. Briefly, cells were grown at 37°C in 125 mL shake flasks containing 25 mL of LB media supplemented with 50 μg/mL kanamycin to mid-exponential phase (*A*_600 _≈ 0.45). Cultures were supplemented with all-*trans *retinal to a 10 μM final concentration and MP synthesis initiated by addition of 0.2% L-arabinose. Cells (5 mL) were harvested 3 h post-induction, disrupted with a French press operated at 10,000 psi, subjected to centrifugation at 10,000 *g *for 10 min at 4°C to remove aggregated material, and the supernatant was centrifuged at 150,000 *g *for 1 h at 4°C to collect membrane material. Samples corresponding to identical amounts of cells (based on *A*_600_) were analyzed by SDS-PAGE and immunoblotting with anti-6-His antibodies (Covance) as described [5]. To collect HtdR adsorption spectra, membranes fractions prepared as above were resuspended in 50 mM 2-(N-morpholino) ethanesulfonic acid (MES), pH 6.5, 300 mM NaCl, 5 mM imidazole, and 1.0% n-dodecyl β-D-maltoside (DDM) and spectra were acquired on a Beckman coulter DU640 spectrophotometer.

### Real-time RT-PCR

RNA was purified from cells grown and induced as above and 1 h after induction of HtdR synthesis using the Aurum Total RNA Mini Kit (BioRad). Total RNA concentrations for each sample were estimated using *A*_260 _measurements and 50 ng RNA was used as template with the iScript One-Step RT-PCR Kit with SYBR Green (BioRad). The reverse transcription (RT) of mRNA encoding HtdR and real-time PCR reactions were done with 8 replicates for each sample, and 16s RNA as the internal standard. Primers 5'-GTGATCGGAAAATGCAGGAG-3' and 5'-GCGATCGTGTTTCGGTTCG-3' were used to quantify mRNA encoding HtdR. Primers 5'-GCCATAACGTCGCAAGACCAAAG-3' and 5'-TTCTTCATACACGCGGCATGG-3' were used for the 16s RNA standard [27]. Relative expression levels were analyzed using the 2^-ΔΔCT ^method [28].

## Competing interests

The authors declare that they have no competing interests.

## Authors' contributions

BLN and FB designed the experiments and wrote the manuscript. BLN performed all experiments. All authors read and approved the final manuscript.

## References

[B1] Wallin E von Heijne G Genome-wide analysis of integral membrane proteins from eubacterial, archaean, and eukaryotic organisms Protein Science 1998 7 4 1029 1038 9568909 10.1002/pro.5560070420 PMC2143985

[B2] Baneyx F Recombinant protein expression in *Escherichia coli* Curr Opin Biotechnol 1999 10 5 411 421 10.1016/S0958-1669(99)00003-8 10508629

[B3] Wagner S Baars L Ytterberg AJ Klussmeier A Wagner CS Nord O Nygren PA van Wijk KJ de Gier JW Consequences of membrane protein overexpression in *Escherichia coli* Mol Cell Proteomics 2007 6 9 1527 1550 10.1074/mcp.M600431-MCP200 17446557

[B4] du Plessis DJ Nouwen N Driessen AJ The Sec translocase Biochim Biophys Acta 2011 1808 3 851 865 10.1016/j.bbamem.2010.08.016 20801097

[B5] Nannenga BL Baneyx F Reprogramming chaperone pathways to improve membrane protein expression in *Escherichia coli* Protein Science 2011 20 8 1411 1420 10.1002/pro.669 PMC3189526 21633988

[B6] Miroux B Walker JE Over-production of proteins in *Escherichia coli: *mutant hosts that allow synthesis of some membrane proteins and globular proteins at high levels J Mol Biol 1996 260 3 289 298 10.1006/jmbi.1996.0399 8757792

[B7] Wagner S Klepsch MM Schlegel S Appel A Draheim R Tarry M Hogbom M van Wijk KJ Slotboom DJ Persson JO Tuning *Escherichia coli *for membrane protein overexpression Proc Natl Acad Sci USA 2008 105 38 14371 14376 10.1073/pnas.0804090105 18796603 PMC2567230

[B8] Studier FW Use of bacteriophage T7 lysozyme to improve an inducible T7 expression system J Mol Biol 1991 219 1 37 44 10.1016/0022-2836(91)90855-Z 2023259

[B9] Guzman LM Belin D Carson MJ Beckwith J Tight regulation, modulation, and high-level expression by vectors containing the arabinose P_BAD _promoter J Bacteriol 1995 177 14 4121 4130 7608087 10.1128/jb.177.14.4121-4130.1995 PMC177145

[B10] Ren H Yu D Ge B Cook B Xu Z Zhang S High-level production, solubilization and purification of synthetic human GPCR chemokine receptors CCR5, CCR3, CXCR4 and CX3CR1 PloS one 2009 4 2 e4509 10.1371/journal.pone.0004509 19223978 PMC2637981

[B11] Romantsov T Battle AR Hendel JL Martinac B Wood JM Protein localization in *Escherichia coli *cells: comparison of the cytoplasmic membrane proteins ProP, LacY, ProW, AqpZ, MscS, and MscL J Bacteriol 2010 192 4 912 924 10.1128/JB.00967-09 20008071 PMC2812954

[B12] Collinson I Breyton C Duong F Tziatzios C Schubert D Or E Rapoport T Kuhlbrandt W Projection structure and oligomeric properties of a bacterial core protein translocase EMBO J 2001 20 10 2462 2471 10.1093/emboj/20.10.2462 11350935 PMC125464

[B13] Ogden S Haggerty D Stoner CM Kolodrubetz D Schleif R The *Escherichia coli *L-arabinose operon: binding sites of the regulatory proteins and a mechanism of positive and negative regulation Proc Natl Acad Sci USA 1980 77 6 3346 3350 10.1073/pnas.77.6.3346 6251457 PMC349612

[B14] Hollands K Busby SJ Lloyd GS New targets for the cyclic AMP receptor protein in the *Escherichia coli *K-12 genome FEMS Microbiol Lett 2007 274 1 89 94 10.1111/j.1574-6968.2007.00826.x 17608696

[B15] Harman JG Allosteric regulation of the cAMP receptor protein Biochim Biophys Acta 2001 1547 1 1 17 10.1016/S0167-4838(01)00187-X 11343786

[B16] Liu-Johnson HN Gartenberg MR Crothers DM The DNA binding domain and bending angle of *E. coli *CAP protein Cell 1986 47 6 995 1005 10.1016/0092-8674(86)90814-7 3536129

[B17] Schultz SC Shields GC Steitz TA Crystal structure of a CAP-DNA complex: the DNA is bent by 90 degrees Science (New York, NY 1991 253 5023 1001 1007 10.1126/science.1653449 1653449

[B18] Schleif R Regulation of the L-arabinose operon of *Escherichia coli* Trends Genet 2000 16 12 559 565 10.1016/S0168-9525(00)02153-3 11102706

[B19] Kamo N Hashiba T Kikukawa T Araiso T Ihara K Nara T A light-driven proton pump from *Haloterrigena turkmenica*: functional expression in Escherichia coli membrane and coupling with a H+ co-transporter Biochem Biophys Res Commun 2006 341 2 285 290 10.1016/j.bbrc.2005.12.181 16413498

[B20] Puertas JM Nannenga BL Dornfeld KT Betton JM Baneyx F Enhancing the secretory yields of leech carboxypeptidase inhibitor in *Escherichia coli*: influence of trigger factor and signal recognition particle Protein expression and purification 2010 74 1 122 128 10.1016/j.pep.2010.06.008 20600941

[B21] Crooks GE Hon G Chandonia JM Brenner SE WebLogo: a sequence logo generator Genome research 2004 14 6 1188 1190 10.1101/gr.849004 15173120 PMC419797

[B22] Parkinson G Wilson C Gunasekera A Ebright YW Ebright RE Berman HM Structure of the CAP-DNA complex at 2.5 angstroms resolution: a complete picture of the protein-DNA interface J Mol Biol 1996 260 3 395 408 10.1006/jmbi.1996.0409 8757802

[B23] Ebright RH Cossart P Gicquel-Sanzey B Beckwith J Molecular basis of DNA sequence recognition by the catabolite gene activator protein: detailed inferences from three mutations that alter DNA sequence specificity Proc Natl Acad Sci USA 1984 81 23 7274 7278 10.1073/pnas.81.23.7274 6390433 PMC392128

[B24] Ebright RH Cossart P Gicquel-Sanzey B Beckwith J Mutations that alter the DNA sequence specificity of the catabolite gene activator protein of E. coli Nature 1984 311 5983 232 235 10.1038/311232a0 6090927

[B25] Shimono K Iwamoto M Sumi M Kamo N Functional expression of *pharaonis phoborhodopsin *in *Escherichia coli* FEBS Lett 1997 420 1 54 56 10.1016/S0014-5793(97)01487-7 9450549

[B26] Datsenko KA Wanner BL One-step inactivation of chromosomal genes in *Escherichia coli *K-12 using PCR products Proc Natl Acad Sci USA 2000 97 12 6640 6645 10.1073/pnas.120163297 10829079 PMC18686

[B27] Feng Y Cronan JE Overlapping repressor binding sites result in additive regulation of *Escherichia coli *FadH by FadR and ArcA J Bacteriol 2010 192 17 4289 4299 10.1128/JB.00516-10 20622065 PMC2937390

[B28] Livak KJ Schmittgen TD Analysis of relative gene expression data using real-time quantitative PCR and the 2(-Delta Delta C(T)) Method Methods (San Diego, Calif 2001 25 4 402 408 10.1006/meth.2001.1262 11846609

[B29] Schneider TD Stephens RM Sequence logos: a new way to display consensus sequences Nucleic Acids Res 1990 18 20 6097 6100 10.1093/nar/18.20.6097 2172928 PMC332411

[B30] Robison K McGuire AM Church GM A comprehensive library of DNA-binding site matrices for 55 proteins applied to the complete *Escherichia coli *K-12 genome J Mol Biol 1998 284 2 241 254 10.1006/jmbi.1998.2160 9813115

